# Assessment of identity development and identity diffusion in adolescence - Theoretical basis and psychometric properties of the self-report questionnaire *AIDA*

**DOI:** 10.1186/1753-2000-6-27

**Published:** 2012-07-19

**Authors:** Kirstin Goth, Pamela Foelsch, Susanne Schlüter-Müller, Marc Birkhölzer, Emanuel Jung, Oliver Pick, Klaus Schmeck

**Affiliations:** 1 Child and Adolescent Psychiatric Hospital, Psychiatric University Hospitals Basel, Basel, Switzerland; 2 Weill Medical College of Cornell University, New York, USA; 3 Practice for Child and Adolescent Psychiatry, Frankfurt/Germany, and University of Applied Sciences FHNW, Basel, Switzerland; 4 Faculty of Medicine, Justus Liebig University, Giessen, Germany

**Keywords:** Identity, Questionnaire, Overview, Psychometrics, Personality disorder, Adolescence

## Abstract

**Background:**

In the continuing revision of Diagnostic and Statistical Manual (*DSM-V*) “identity” is integrated as a central diagnostic criterion for personality disorders (self-related personality functioning). According to Kernberg, identity diffusion is one of the core elements of borderline personality organization. As there is no elaborated self-rating inventory to assess identity development in healthy and disturbed adolescents, we developed the *AIDA* (Assessment of Identity Development in Adolescence) questionnaire to assess this complex dimension, varying from “Identity Integration” to “Identity Diffusion”, in a broad and substructured way and evaluated its psychometric properties in a mixed school and clinical sample.

**Methods:**

Test construction was deductive, referring to psychodynamic as well as social-cognitive theories, and led to a special item pool, with consideration for clarity and ease of comprehension. Participants were 305 students aged 12–18 attending a public school and 52 adolescent psychiatric inpatients and outpatients with diagnoses of personality disorders (N = 20) or other mental disorders (N = 32). Convergent validity was evaluated by covariations with personality development (*JTCI 12*–*18 R* scales), criterion validity by differences in identity development (*AIDA* scales) between patients and controls.

**Results:**

*AIDA* showed excellent total score (Diffusion: α = .94), scale (Discontinuity: α = .86; Incoherence: α = .92) and subscale (α = .73-.86) reliabilities. High levels of Discontinuity and Incoherence were associated with low levels in Self Directedness, an indicator of maladaptive personality functioning. Both *AIDA* scales were significantly different between PD-patients and controls with remarkable effect sizes (d) of 2.17 and 1.94 standard deviations.

**Conclusion:**

*AIDA* is a reliable and valid instrument to assess normal and disturbed identity in adolescents. Studies for further validation and for obtaining population norms are in progress and may provide insight in the relevant aspects of identity development in differentiating specific psychopathology and therapeutic focus and outcome.

## Background

Identity and its disturbance are viewed as central constructs in psychoanalytic and psychodynamic theories, finding its counterparts in the area of social-cognitive theories using terms such as basic “self-concepts” and “mental representations”. In general terms, identity could be defined as ,,unity of being” but the attempt to find a comprehensive definition immediately shows its hybrid nature, being both intrapsychic and interpersonal, and its various phenomenological aspects complicating an operationalization along its true constituents [1].

In the following, we will discuss first concepts of healthy identity development and then concepts of disturbed identity, both times addressing psychodynamic as well as social-cognitive and empirical approaches. With this background, we will motivate the concrete scale development in contrast to perceived shortcomings of existing approaches.

Erikson described identity as a fundamental organizing principal, developing constantly throughout life and providing a sense of continuity within the self and in interaction with others (,,self-sameness“) as well as a frame to differentiate between self and others (,,uniqueness“), which allows the individual to function autonomously from others [2]. He described the consolidation of identity as a central task in normal adolescent development, when previous identifications and introjections had to be shed and transformed in a process that is called an identity crisis. In the operationalized psychodynamic diagnostic inventory (*OPD-2)*, normal identity is described as ,,… the entirety of the inner pictures of oneself”, closely related to the “ideal self”. In its development “special phases lead to conflicts that may result in a subjective feeling of continuity and coherence, when integration of new self-images into identity succeeds.“ [3]. As a result, a stable identity plays a role in self-esteem, a realistic appraisal of self and others, and insight into the effect one has on another [4]. Therefore, identity aids in self-reflective functioning, autonomy, effective social exchanges and provides predictability and continuity of functioning within a person, across situations, and across time [5].

A distinction between two different aspects of identity can be found in many theories from social-cognitive and developmental psychology [6,7]. James (1890 in [6]) made the classical distinction between the “I”, an intuitive, emotionally experienced vital self-evidence, and the “ME”, a result of a self-reflective process leading to an integrated awareness and knowledge about oneself. Thus, identity can be divided into the two higher order domains “subjective self” (focussing on continuity, “stable core”, emotional access) and “definitory self” (focussing on coherence, “integrated whole”, cognitive access). In contrast, Stern (1985 in [6]) postulated four components of self: “self-agency” (sense of authorship) and “self-coherence” (sense of non-fragmented, physical whole with boundaries) as well as “self-affectivity” (experiencing inner qualities of feeling) and "self-history" (,,going on being'', the possibility to change while remaining the same). Different authors introduced different sets of single self-concepts to fully describe a person’s “identity system”. Bracken [8] articulated six self-concepts which refer to different areas of psychosocial functioning: Social, Competence, Affect, Academic, Family, and Physical. Deusinger [9] describes ten self-concepts reflecting: efficiency, problem solving, certainty in behavior- and decision making, self worth regulation, sensibility and moodedness, persistence, social ability, appreciation from others / role security, confusability, emotions and relationships. Referring to Erikson’s concept of ego growth, strength and synthesis [10], Marcia [11,12] differentiates between the four statuses of identity formation: Diffusion, Foreclosure, Moratorium and Achievement. Each formation is defined by a specific combination of high vs. low “commitment” and “exploration”, regarded as the central areas for defining identity. Associated approaches strengthen the necessity of a cognitive elaboration of commitments to constitute identity achievement, which is linked to a healthy development [13,14].

Fonagy et al. [15] combined psychoanalytic concepts with attachment theory and ,,theory of mind'' to a joint concept of ,,mentalization'', describing the development of complex mental representations of self and others based on the development of emotion regulation (self-control, affect-control), the capacity for intersubjectivity (imitation, role-acceptance, change of perspective), and reflective self-functions. These mental representations evolve progressively as a result of self-reflection and facilitate the understanding, prediction, and consideration of ones own and others' mental states. This can be viewed as a basic requirement for the formation of an experience of identity. Additionally, Seiffge-Krenke [16] emphasizes the significant changes in adolescence, not only by the need to develop entirely new self-images and roles (e.g. as a sexual partner), but also by the age-related cognitive changes from concrete to formal operational patterns (abstract) of thinking and by the need to “debond” from the parents. This creates feelings of loneliness, sadness, anger and emotional detachment and an "erosion" of the former stabilizing child's identity.

According to Otto Kernberg, identity crisis results from the discrepancy between rapidly shifting physical and psychological experiences, on the one hand, and a widening gap between self-perception and the experiences of others’ perceptions of the self, on the other hand [17]. In identity crisis, continuity of self remains across situations and across time despite experimentations with different roles and usually resolves into a normal, consolidated identity with flexible and adaptive functioning [5]. This permits the adolescent or young adult to develop rewarding and satisfying friendships, to form clear life goals, to interact appropriately with parents and teachers, to establish sexual and intimate relations, and to develop positive self-esteem [18].

In contrast, identity diffusion is viewed as a lack of integration of the concept of self and significant others. This results in a loss of capacity for self-definition and commitment to values, goals, or relationships, and a painful sense of incoherence. This is often observed as “unreflective, chaotic and contradictory descriptions of the patient about himself and others” and the “inability to integrate or even perceive contradictions” [19,20]. According to Kernberg, an incompletely integrated identity may additionally manifest in either chronic emptiness, contrary behavior and superficiality or in other signs of weak ego-strength like poor anxiety tolerance and impulse control. Identity development can be described as a continuum with an identity diffusion (incoherent self-image, self-fragmentation) at one end and an integrated personal identity at the other end [21]. Overall, identity diffusion is a core element of the “borderline personality organization” [21] and is viewed as the basis for subsequent personality pathology, leading to a broad spectrum of maladaptive and dysfunctional behaviors [14].

Other authors focus on borderline personality disorder (BPD) in their studies, since this patient group characterizes significant personality pathology particularly in the disturbance of identity. Westen described “identity disturbance” as the central construct for detecting severe personality pathology, and most notably BPD, in adults and adolescents, containing the dimensions: lack of commitment, role absorption, painful incoherence and lack of consistency, assessed with an expert rated questionnaire *IDQ*[22]; Crick developed a questionnaire (*BPFS-C)* to assess borderline personality features in children, based on Morey’s concept for adults, which integrates “identity problems” in addition to the factors affective instability, negative relationships and self-harm [23]. Poreh established a *DSM-IV* criteria based questionnaire (*BPQ)* to assess borderline personality in adults with nine subscales: Impulsiveness, Affective Instability, Abandonment, Relationship, Self-Image, Suicide/Self-Mutilation, Emptiness, Intense Anger, and Quasi-Psychotic States, all contributing empirically to a joint borderline factor called “Identity/Interpersonal” [24,25]. In the *DSM-IV*[26] identity disturbance (i.e. “markedly and persistently unstable self-image or sense of self,” p. 654) is included as one of the components of borderline personality disorder. This was supported empirically by many findings, including Becker [27] who found identity disturbance and affective dysregulation in adolescents to be the most significant symptoms in leading to a correct diagnosis of borderline personality disorder.

The lack of empirical support for the categorical method of diagnosing personality disorders, diagnostic thresholds and the heterogeneity of PD diagnoses [28,29], led to a complete revision [30] of PD diagnoses for the new *DSM-V* (http://www.dsm5.org). From 2013 on, a hybrid model including dimensions and categories shall be used. At present, six specific personality disorder types (antisocial, schizotypal, borderline, narcissistic, obsessive-compulsive, avoidant) should be evaluated according to a set of criteria based on core impairments in personality functioning and pathological personality traits from two different domains: self functioning (dysfunctionality) and interpersonal (social maladaptivity). Impairments in self functioning are reflected in dimensions of identity and self-direction. Interpersonal impairments consist of impairments in the capacities for empathy and intimacy. With this, the concept of identity per se and Kernberg’s concept of identity diffusion is assigned to play a central role in defining and detecting personality disorders on a general level, not only as a specific trait in borderline PD. As inventories and interviews for assessing the new criteria are under construction internationally, also identity has to be modeled in a highly structured and elaborated way.

Early signs of personality disorders, with considerable stability despite developmental stage [31-33], are apparent before the age of 18 [34,35]. Therefore, deviations from normal personality development in children and adolescents can and should be identified and targeted for intervention [5,22,36,37]. As adolescent identity diffusion can be described consistently with Otto Kernberg’s conceptualization of adult identity diffusion [38,39], the treatment designed for adults with identity diffusion *TFP* (Transference Focused Psychotherapy) [40] should be effective in adolescents with identity diffusion as well, provided that developmentally appropriate modifications are implemented. Paulina Kernberg elucidated in 2000 a model for understanding identity pathology in children and adolescents and postulated that identity diffusion is the result of failure to consolidate identity at each stage from childhood through adolescence [5]. Her emphasis in adolescence was on the need to differentiate those with normal identity crisis from those with identity diffusion and to intervene directly during this developmental period. In this sense, and in continuing the work of Paulina Kernberg, the psychotherapeutic approach *TFP-A* (Transference Focused Psychotherapy - Adolescent Identity Treatment, AIT) [4,41] was developed to treat adolescents with identity diffusion in order to help them to improve identity integration and hence increase adaptive functioning and behavior by improving their relationships with friends, parents, and teachers, acquiring positive self-esteem, clarifying life goals and be better prepared for entering love relationships [18,42].

Based on the concepts described above, our Swiss-German-American research group started in 2010 to develop the questionnaire *AIDA* (Assessment of Identity Development in Adolescence) to measure identity development in adolescents. *AIDA* is designed to overcome psychometric shortcomings of the questionnaire *IPO-CH*[43], an adaption of the *IPO*[44] (“Inventory for Personality Organization”) for children and adolescents. For example, the heterogeneity of the scales and the ambiguity and confounds with non-target constructs like trait-impulsivity on the item level [45]. The construct “identity” has been given the priority over other disturbance-related aspects like object relations, primitive defences, moral values, aggression or reality testing. These have been integrated relative to their relation to identity diffusion. Following this approach, the development of an adapted version for adolescents of the interview *STIPO*[46] is currently in progress by an Italian research-group.

### Scale construction for *AIDA*

Our initial goal was to assess identity development on a well-founded Likert scale ranging from “healthy” to “disturbed” in order to differentiate healthy identity development from a current identity crisis as well as from a severe identity diffusion. This was part of our research about the prevalence and specific development of personality disorders in adolescence. But our review of literature yielded that the existing approaches were either too much focused on pathology and did not assess normal variants of identity development adequately or they focused on healthy development and disregard a structured integration of disturbed personality. The former were mostly formulated in interview form [46] or as an expert rating [22], symptom-oriented in content and, even as a self- rating questionnaire [47], usually targeting adults. The latter are predominantly developed as self-rating questionnaires, similar to personality inventories, and designed to capture general self concepts without specifying an elaborated link to pathology [8,9,48,49], even in Akhtar & Samuel’s *ICI* to assess explicitly “components of identity” [50]. So we decided to develop a new questionnaire based on a broad description of the field, using a deductive test construction, in which the structure of a targeted construct is carefully elaborated with respect to underlying factors concerning causation, psychological, or social functions [51,52], and following strict modeling techniques concerning the internal structure of higher order scales, subscales and facets with precise definitions within (truly shared content) and differentiations between them (no shared content or trivial item-overlap) [53,54] to maximize construct validity. For conceptual clarification and a broad capturing of normal as well as disturbed development of identity, the scale construction process for *AIDA* integrated the concordant approaches from psychoanalytic and social-cognitive psychology (see above) and, additionally, the constructs, subconstructs, and items modeled by existing inventories for assessing identity had been analyzed carefully and integrated in a re-assembled way. In this process, we kept the originally used names for the subconstructs as far as possible to facilitate traceability and clarity of the content.

From the abovementioned theoretical descriptions about identity development, two domains could clearly be distinguished in line with the constructs´ dichotomy in social-cognitive psychology as well as in the psychopathology-oriented psychodynamic descriptions: a basic distinction between “Continuity” and “Coherence”, serving as a well elaborated theoretical framework to find a meaningful and distinct substructure of the higher order construct “identity integration vs. identity diffusion”.

· The construct “Continuity” represents the vital experience of “I” and subjective emotional self-sameness with an inner stable time line. High “Continuity” is associated with the stability of identity-giving goals, talents, commitments, roles, and relationships, and a good and stable access to emotions as well as the trust in the stability of them. A lack of Continuity (i.e. high “Discontinuity”) is associated with a missing self-related perspective, no feeling of belonging and affiliation, and a lack of access to emotional levels of reality and trust in the durability of positive emotions.

· The construct “Coherence” stands for clarity of self-definition as a result of self-reflective awareness and elaboration of the “ME”, accompanied by consistency in self images, autonomy and Ego-strength, and differentiated mental representations. A lack of Coherence (i.e. high “Incoherence”) is associated with being contradictory or ambivalent, suggestible and over-matching, and having poor access to cognitions and motives, accompanied by superficial and diffuse mental representations.

**Figure 1:**
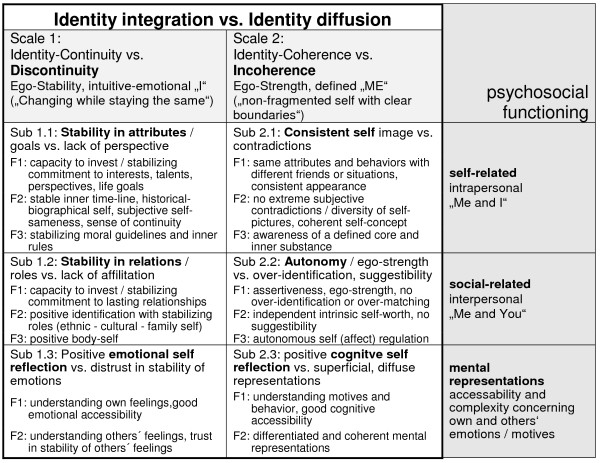
**Theory-based suggestion for a meaningful substructure of the construct “Identity Integration vs Identity Diffusion” and its operationalization into  *AIDA * scales, subscales, and facets.**

Within these two domains, we additionally subdivided each into three different sub-domains, each reflecting the different areas of psychosocial functioning: self-related, social-related, and ability/reflection-related (see Figure 1). This enabled the reassemblance of the known identity-related subconstructs into a meaningful joint framework, providing a maximum of source-related compilation of the contents based on the theoretical descriptions. With this, we are uniting the “hybrid nature” of the construct (being both intrapsychic and interpersonal, [1]), the studies related to developmental identity formation (distinct aspects commitment and exploration) [12], and concepts of identity-related reflective functioning and mental representations according to Fonagy [15,47] in an elaborated way. To a great extent, we could integrate the central operationalizations of identity diffusion (ID) by O. Kernberg (capacity to invest, continuity over time, representation of others, superficiality, loneliness, self-coherent opinions and self esteem) [21] and Westen (lack of commitment, role absorption, over-identification, painful ambivalence, inconsistency) [22] into the described higher-order structure. Compared to the described “levels of personality functioning” for the *DSM-V*, all central aspects of identity are integrated in the *AIDA* structure as well.

The construction process of the concrete item formulations to integrate the referred subconstructs addressed a central shortcoming of some of the existing inventories: the lack of clarity concerning the targeted subconstructs (e.g. mixed contents) and/or the inappropriateness of the formulations for self-assessment in adolescents (e.g. too complicated).

The complexity of construct clarification in test operationalization is showing clearly within the aspect “identity related to relationships“. On the one hand, the adoption of and identification with social roles, such as in the family, sexual roles, and cultural roles, is stabilizing identity in a very positive way, fully corresponding with Samuel and Akhtars´ components of identity and in our model assigned to the area Continuity. But, on the other hand, a too strong identification with roles and openness for social attention is seen as a sign of identity disturbance called e.g. role-absorption and over-identification in Westen’s concept and described as not having own opinions, goals, and self-esteem, being defined by others, which is in our model clearly assigned to the area Coherence. The difference lies in the true integratedness of the adopted roles and if they really match with one’s talents and perspectives or if they are just an artificial mask, the latter speaking for a lack of autonomy and assertiveness against social influences. It is obvious that this difference is highly significant and can not be assessed by asking the number of roles a person is identified with, as a lot of roles may indicate either a positive or negative sign concerning identity development. So we tried to keep out all mixed or unclear contents and targeted directly either “Continuity – stabilizing roles vs. lack of social roots” or “Coherence – autonomy vs. suggestibility” in our test construction.

Similarly, we tried to make clear the distinction concerning ,,identity disturbance in terms of being contrary – or being unstable – or experiencing painful ambivalence“. In simple terms: it makes a tremendous difference concerning assumed identity integratedness if an adolescent is switching hobbies and life goals because of (a) having an impulsive temperament or (b) having a lack of internal temporal continuity to himself, his social environment and his feelings (self-sameness) or (c) having different hobbies with every different peer group like a chameleon while the different self’s are not connected on a higher level (self-coherence). To catch the truly targeted construct “identity” it is crucial to separate the distinct subconstructs regarding their clinical and psychological impact (e.g. “unsettled, not-persistent“ vs. “chaotic, empty, two-faced“), even though it may look the same from the outside (phenotype “switching hobbies”) and to leave out the non-target constructs in the scale and item construction process, especially “impulsivity”. Trait impulsivity itself is not regarded as a risk factor to develop a personality disorder and may just be used to characterize the type, if a personality disorder should occur throughout life. Given this, it is crucial to keep out any impulsivity items to catch the phenomenon “identity” with reference to a disturbed development. Impulsivity, as a quasi-automatic emotional tendency to change interests and hobbies, to make quick decisions, to react before thinking, and to be prone to sensation seeking, can thus be seen as a perfect alternative hypothesis to what is described as “identity discontinuity” in terms of being unsure about own talents, own feelings, own affiliations. To summarize, being impulsive can be fun and lively and experienced as an active “I” whereas, having no inner continuity is not.

Altogether, the inventory *AIDA* is substructuring the higher order construct “Identity Diffusion” as constituted by the two separable scales “Disontinuity” and “Incoherence”, each assessed as a sum of their three subscales reflecting distinct psychosocial functions. The facet level presented in Figure 1 is not supposed to be used independently (i.e. like sub-subscales) but is defined to facilitate conceptual clarity and to ease stringent scale and item construction. All scales are coded towards pathology, so high scores indicate high disturbance.

This current study examines the psychometric properties of the questionnaire *AIDA*. The sufficiency of homogeneity is tested by several item coefficients, scale reliabilities Cronbach’s α, and phenotypical factorial structure in explorative factor analyses (EFA). The construct validity is examined by convergent and discriminant validities with related constructs, here with the personality dimensions according Cloninger’s biopsychosocial model, and the construct validity, in terms of diagnostic validity, is evaluated directly by comparing the *AIDA* scores on scale and subscale level between psychiatric patients and healthy controls.

Cloninger’s biopsychosocial model of personality claims to provide insight in the development of personality disorders as well as giving a theory-based and elaborated description of overall personality [55-58]. By dividing the two areas of personality “temperament” from “character” it combines person-centered aspects of general vulnerability and environment-centered aspects of dysfunctional influences and allows the evaluation of an individual’s current maturity in terms of impaired personality functioning. Thus, Cloninger’s model is ideally suited for investigating PD-related issues [59-62]. With the JTCI-R-family (Junior Temperament and Character Inventory) the concept can be assessed by questionnaire in adolescents (12–18 years) equivalent to the revised adult version *TCI R* with excellent results for reliabilities and validity [63,64]. With its two central diagnostic factors Self Directedness and Cooperativeness, Cloninger’s concept of character perfectly covers the new *DSM-V* criteria concerning PD diagnoses. Especially the herein described impairment of intrapersonal personality functioning is supposed to be covered by the combination of Self Directedness (*JTCI 12–18 R)* and Identity Diffusion measured by *AIDA*.

## Methods

### Participants and Procedures

We assessed a clinic and a school sample to (a) gain a heterogeneous sample for test validation by mixing children and adolescents with typical development and those with assumed identity problems in order to cover the whole distribution of the targeted construct and avoid sample-specific ceiling or floor effects that potentially distort item-characteristics and to (b) provide data for analyzing the criterion validity and detailed relations to specific psychopathology of the *AIDA*-scores by comparing the results of patients and healthy controls. The study was approved by the Ethics Committee Basel / Switzerland (EKBB) as well as by the Ministry of Education Hessen / Germany.

Sample I consisted of 305 6–12 grade adolescent students (148 boys, 157 girls) from two public schools which were chosen as representative of the area. The mean age of the sample was 15.00 years (SD 2.01), age range was 12 to 18 years. Data collection took place at the schools in a group-setting by classes or grades during one school hour. Prior to the assessment the study was explained to the students and written consent from the parents, that had been handed out one week before, was collected as a requirement for participation. In a classroom setting, with an undergraduate research assistant available to answer questions, the students were asked to fill out the two questionnaires by themselves without talking. The total classroom participation rates ranged from 63% to 86% (MEAN = 74%).

Sample II involved a clinical sample of 52 adolescents (17 boys, 35 girls), with ages ranging from 12 to 18 years and a mean age of 15.58 years (SD *=* 1.83). Participants were inpatients and outpatients of a child and adolescent psychiatric university hospital and a child and adolescent psychiatric practice. Inclusion criteria were age 12–18 years, sufficient linguistic and cognitive skills to master the written task and no current psychotic episode. The patients showed a variety of psychiatric problems, N = 20 with diagnoses of personality disorders (N = 18 type “emotional-unstable”), N = 12 with affective disorders (anxiety, depression), N = 7 with attention and conduct disorders and N = 13 showing high comorbidity. Diagnoses were based on clinical interviews (see below). Following the approved IRB protocol, therapists provided a complete description of the study to the participants and written informed consent was obtained from the adolescents and the parents. The two semi-structured interviews were conducted by a graduate psychological research assistant.

### Measures

#### *AIDA*

*AIDA* was developed following systematic test construction procedures [65] with two stages. First stage was the theoretical explication of the targeted construct and the generation of a specific initial item pool by expert consensus. These items were pretested to ensure ease of comprehension and clarity of the items in the targeted age group. This served as the basis for further item modifications. Second stage was the empirical selection based only on the obtained statistical or psychometric properties of the items in the main sample to derive the final item pool and establish the targeted scales. Following this, all *AIDA* items were reviewed in detail between the authors, introducing different approaches and expertises, to obtain final consensus agreement. We focused on the items´ conceptual distinctness and each definite relation to pathological or healthy identity development as well as on their true potential to be answered correctly by adolescents concerning effects like social desirability, gender-related bias and conscious accessibility of the content (e.g. the statement “I admire people in order to feel secure” may be asked by an expert in an interview-situation, but would pose validity concerns in self-rating). The latter involves special considerations about age-related ability for self-reflection and/or the emotional discomfort, especially regarding sexual issues in a questionnaire-situation without having a relationship to the investigator. While the topic is clinically relevant, a component of identity and a phenotypical marker of the construct, it was omitted from the item pool due to the lack of reliability and validity in a self-report format. Thus, this important topic “concrete sexuality – gender-related satisfaction” will need to be evaluated by the therapist, as simply not every issue is applicable to this kind of operationalization.

The initial item pool with 102 items had been tested with 15 adolescents, leading to some modifications and a reduced pilot version with 96 items (e.g. leaving out the items about sexual development because of high missing rates or negative feedback of the adolescents). Items were rated on a 5-point Likert scale (0 = no, 1 = more no, 2 = part/part, 3 = more yes, 4 = yes). Additionally, six semi-open questions about own and best friends´ hobbies or interests (e.g. “What kind of hobbies or interests do you have, that describe you well?”), perceived group-affiliations, and typical attributes were asked to challenge the probands productivity and simulate an interview-like situation for creating a set of supportive variables in expert rating, using a fixed coding schema. These variables focus on contents that are difficult to catch with classical items, on the one hand covering the *AIDA* facets “superficiality vs. differentiated descriptions / representations” and “over-identification”, on the other hand integrating two new subconstructs “self-stigmatizing” (following Westen [22]) and “compliance vs. defiant attitude”. This *AIDA* pilot version had been tested with 47 adolescents aged 11–19 (MEAN 15.51, SD 2.39; 62 % girls), enriched with the first 22 patients (12 with PD diagnosis) of our clinical sample (age MEAN 15.86, SD 1.89; 64 % girls) and a preliminary testwise item-selection with this N = 69 sample supported a fully reliable reduced questionnaire with the suggested scale structure and reliabilities of α ≥ .90.

#### *JTCI 12–18 R*

*JTCI 12–18 R*[63] (Junior Temperament and Character Inventory - Revised) contains 103 statements in a five-step answer mode to assess personality development via four temperament scales (“Novelty Seeking / behavioral activation”, “Harm Avoidance / behavioral inhibition”, “Reward Dependence / social responsiveness”, “Persistence / intrinsic motivation”) and three character scales (“Self Directedness / individual functionality”, “Cooperativeness / social adaptivity”, “Self Transcendence / embeddedness”) in self-rating according to Cloninger’s biopsychosocial model and is appropriate for adolescents between 12–18 years (+/− 2 years). It is part of a test set constructed in German language in cooperation with Cloninger to reflect his revised operationalization for adults (*TCI R*) [66] on truly equivalent scales for children (*JTCI 3–6 RJTCI 7–11 R)* and adolescents (*JTCI 12–18 RJTCI 12–18 R Parent)* on scale and defined subscale level [67]. Psychometric properties for all these JTCI-R versions are very good [67,68], for the German *JTCI 12–18 R* the scale reliabilities α are between .79 and .85, excellent construct validity had been shown with CFA (temperament: *CHI*^2^/df: *CHI*^2^/df = 1.96, *RMSEA* = .05, *AGFI* = .96; character: *CHI*^2^/df: *CHI*^2^/df = 0.43, *RMSEA* = .00, *AGFI* = .99) [64] and promising results for diagnostic validity were demonstrated by assumed covariations with severity (character scales) and type (temperament scales) of current psychopathology [67].

### ** *SCID-II* ** and ** *K-DIPS* **

As the aim was to explore the thresholds between healthy development, identity crisis and identity diffusion, valid and broad measures for psychopathology were needed. We used the two well-established semi-structured diagnostic interviews *SCID-II*[69] and *K-DIPS*[70]. *SCID-II* (The Structured Clinical Interview for *DSM-IV* Axis II) is designed to assess personality disorders according to *DSM-IV* criteria. Administration time is about 90 minutes. *K-DIPS* (Children – Diagnostic Interview for Psychiatric Diseases) is designed to assess axis I psychopathology in children and adolescents according to *ICD-10* and *DSM-IV* criteria, and takes about 90–120 minutes to administer.

### Statistical analysis

The Statistical Package for the Social Sciences (SPSS 16 for Windows) was used for data analyses. Item analyses and selection was based on the criteria: percentage of symptomatic answers (5-95%), effect size *f* of gender- or age-related item bias < .40, mean item-total correlation r_it_ > .30, and potentially improving scale reliability Cronbach’s α by item rejection while avoiding trivial redundancy as well as keeping a broad balance of scale content. Therefore, the item selection was carried out subscalewise. The mean r_it_ was built of the results referring to the subscale, the total scale, and the subscale in the clinical subgroup. Additionally, the r_it_ coefficients were analyzed in the subsamples “gender” and “age-group” (see below) and should not be below .20. Scale reliabilities, as a sign of internal construct validity, were evaluated by Cronbach’s α and were supposed to exceed .80 at total scale level, .70 at scale level, and .60 at subscale level as appropriate for heterogeneous contents, while homogeneity coefficients α > .80 would be very good and > .90 excellent [71,72]. In an additional EFA on item level (PCA with varimax rotation to take account for the maximum potential differences between the contents) we examined the phenotypic dimensionality of *AIDA*. Due to the construction we expected a high total congruence, as the scales were not optimized towards statistical independence but towards a joint representation of a complex construct, following basic psychosocial- and pathology-related qualities, which are usually not matching phenotypic correlational patterns.

Construct validity was examined with Pearson correlations between the *AIDA* scales and subscales and should reflect a substantial similarity between the identity-related subconstructs on the one hand (coefficients > .30-.50) but should not reflect a very high similarity (coefficients > .70) on the other hand in order to support the construct’s subdivision.

To assess convergent and discriminant validity, Pearson correlations between *AIDA* and the *JTCI 12–18 R* on scale level were examined with reference to assumed covariances concerning identity diffusion and quality of personality functioning (maturity of character development) and non-covariance concerning basic temperament features, while coefficients should lie between .30 (medium effect size) and .50 (great effect size) to be interpreted substantially in terms of construct validity [73].

In reference to Meeus [13], we divided the sample by age into early-to-middle (12–14 years) and middle-to-late adolescence (15–18 years). Taking into account the results concerning girls reaching more often the identity status “achievement” in the interpersonal identity domain than boys [74] we also analyzed the data separately by gender to identify possible systematic differences in identity structure and development. On the item level, potential gender differences were analyzed by unidimensional ANOVAs to test for inherent item bias that would lead to item rejection and, thus, ensure items are gender neutral. On the scale level, the equivalence of results concerning reliability was evaluated in age- and gender-related subsamples to provide broad appropriateness. In the final step, t-tests on scale level regarding plain score differences between the groups were analyzed and can be interpreted as valid “developmental” group differences, as the other potential influences by age and gender on the results had been excluded empirically in the first and second step of analyses. Score differences had been examined not only concerning significance (1% level) but concerning effect size *d*, conservatively calculated by (AM1-AM2) / ((SD1 + SD2)/2) [73] and were supposed to reach a high amount (>.80) to avoid over-interpretation and artificial establishing of developmental differences. Content validity was analyzed by comparing the *AIDA* results between psychiatric patients with personality disorders (with assumed high amounts of identity diffusion) and healthy controls from the school sample by t-tests.

## Results

### Item selection and scale reliabilities

Item analysis and selection led to a final, 58-item, version of *AIDA* with very good scale reliabilities and a balanced content in line with the theoretical derived model. All remaining items matched the major selection criteria. Concerning the additional selection criteria in the subsamples, only one item (item 40: “I usually have typical ‘on again – off again’ relationships”) showed a remarkably decreased item-total correlation with r_it_ = .09 in the “younger” and r_it_ = .08 in the “male” subsample as a sign of age and gender specifity, while showing sufficient coefficients (.41 in the “older”, .46 in the “females”) in the other subsamples. But as this item is reflecting “romantic relationship” it is not surprising that the younger adolescents did not show similar covariances and we kept the item because of its high impact for stabilizing identity development in the older adolescents.

**Figure 2:**
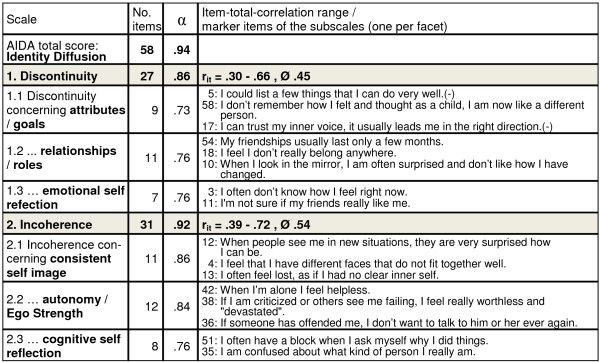
**Scale reliabilities α for the total score, the scales, and the subscales of AIDA in the total sample N = 357, range and medium item-total correlations r**_**it**_** per primary scale and two marker items per subscale. (−) = reverse scoring.**

Reliability coefficients Cronbach’s α were excellent for the total scale Identity-Diffusion with .94, very good for the two primary identity scales Discontinuity and Incoherence with .86 and .92 respectively, and very good for the subscales ranging from .73 to .86. Figure 2 gives a summary of scale and subscale reliabilities, range and medium item-total correlations per primary scale, and marker items per subscale. The results for scale reliabilities were stable in all subsamples (see Table 1) as required for adequate gender and age neutrality on scale level.

**Table 1:** **Differentiated scale reliabilities α and systematic mean score (M) differences with associated effect sizes  *d*  concerning gender (girls N = 192, boys N = 165) and age group (12–14 N = 149, 15–18 N = 208)**

	**gender differences**					**age differences**				
	**Girls**		**Boys**			**12-14**		**15-18**		
	α	M (SD)	α	M (SD)	d	α	M (SD)	α	M (SD)	d
AIDA total score: **Identity Diffusion**	.94	78.12 (32.60)	.93	61.60 (27.51)	**0.55**	.92	**70.85 (28.92)**	.95	70.22 (33.15)	**0.02**
**1. Discontinuity**	.87	32.85 (14.73)	.83	26.74 (12.32)	**0.45**	.82	30.30 (12.91)	.89	29.83 (14.74)	**0.03**
1.1 attributes	.72	14.24 (5.64)	.75	13.00 (6.19)	**0.21**	.70	13.87 (5.91)	.75	13.53 (5.95)	**0.06**
1.2 relationships	.77	8.64 (6.21)	.74	6.44 (5.57)	**0.37**	.69	7.79 (5.69)	.80	7.50 (6.24)	**0.05**
1.3 emotional	.76	9.97 (5.39)	.73	7.30 (4.58)	**0.53**	.73	8.65 (5.22)	.78	8.80 (5.20)	**0.03**
**2. Incoherence**	.91	45.27 (19.64)	.92	34.86 (17.69)	**0.56**	.90	40.55 (18.58)	.93	40.39 (20.09)	**0.01**
2.1 consistent self	.87	16.23 (9.00)	.82	11.47 (7.13)	**0.59**	.82	13.94 (7.90)	.89	14.10 (8.95)	**0.02**
2.2 autonomy	.79	17.06 (7.96)	.84	13.93 (7.72)	**0.40**	.81	15.66 (8.27)	.82	15.58 (7.82)	**0.01**
2.3 cognitive	.74	11.98 (5.65)	.75	9.45 (5.39)	**0.46**	.71	10.95 (5.69)	.80	10.72 (5.65)	**0.04**

**Figure 3:**
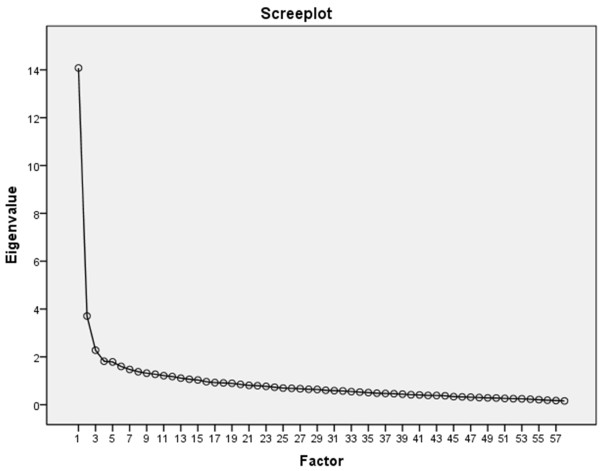
Screeplot for EFA on AIDA item level, 15 extracted components explaining 62.6% variance, first component 24.3%.

In an unrestricted EFA, 15 components were detected that could not be interpreted reasonably in terms of phenotypically independent subscales. While the first component showed an Eigenvalue of 14.08 accounting for 24.27% of the shared variance, the following components only contributed minor explanatory power up to 62.6% in total successively. This speaks for the expected overall congruence on phenotype-level, as all modelled contents/items are supposed to reflect pathology-related identity development but each addressing different aspects. (Figure 3)

### Construct validity

Table 2 shows the intercorrelations of the *AIDA* scales and subscales. As expected, the subscales were highly correlated with their assigned primary scale about .80 but showed lower correlations with each other, as it is required for subsuming scale scores on the one hand and subdividing subscale scores on the other hand. Nevertheless, correlations > .70 occurred between six subscales and the correlation .76 between the two primary scales Incoherence and Discontinuity was higher than expected. Except with the subscale 1.1 “Discontinuity concerning attributes” (.61), the correlations with the total score were about .80 and higher, supporting the appropriateness of an overall sum for “Identity Diffusion”.

**Table 2:** AIDA scale and subscale intercorrelations

	**1.**	1.1	1.2	1.3	**2.**	2.1	2.2	2.3
AIDA total score: **Identity Diffusion**	**.92**	**.61**	**.81**	**.84**	**.96**	**.90**	**.78**	**.83**
**1. Discontinuity**		.78	.87	.80	**.76**	.78	.54	.67
1.1 attributes			.49	.39	.43	**.49**	.26	.37
1.2 relationships				.61	.68	.73	**.46**	.58
1.3 emot. self-refl.					.76	.70	.64	**.70**
**2. Incoherence**						.90	.87	.87
2.1 consistent self							.61	.71
2.2 autonomy								.64
2.3 cogn. self-refl.								

### Discriminant and convergent validity

As expected, all identity -scales and subscales showed high negative correlations with the *JTCI 12–18 R* character scale Self Directedness (−.59 – -.76) but, against our assumptions, only very low correlations with the character scale Cooperativeness (see Table 3). The correlations with the temperament scales were in line with theory, only low toward positive (.03 – .12) with the temperament factor Novelty Seeking / behavioral activation, moderate (mostly < .30) and toward negative with Reward Dependence / social responsiveness (−.01 – -.30) and Persistence (−.08 – -.38) and, displaying the joint relation to psychopathology, substantial positive correlations between identity development (Discontinuity and Incoherence) and Harm Avoidance / behavioral inhibition (.33 – .60) occurred.

**Table 3:** AIDA scale correlations with JTCI 12–18 R scales

	**NS**	**HA**	**RD**	**P**	**SD**	**CO**	**ST**
AIDA total score: **Identity Diffusion**	**.09**	**.59**	**-.21**	**-.23**	**-.78**	**-.09**	**.29**
**1. Discontinuity**	.11	.49	-.30	-.29	-.76	-.15	.18
1.1 attributes	.09	.33	-.31	-.38	-.60	-.27	.00
1.2 relationships	.05	.39	-.31	-.18	-.63	-.11	.20
1.3 emot. self-refl.	.12	.50	-.10	-.13	-.64	.03	.26
**2. Incoherence**	.08	.60	-.12	-.17	-.70	-.04	.33
2.1 consistent self	.08	.48	-.18	-.20	-.66	-.07	.30
2.2 autonomy	.03	.60	-.01	-.08	-.59	.00	.29
2.3 cogn. self-refl.	.10	.49	-.12	-.16	-.59	-.03	.28

NS = Novelty Seeking, HA = Harm Avoidance, RD = Reward Dependence, P = Persistence, SD = Self Directedness, CO = Cooperativeness, ST = Self Transcendence.

### Descriptive statistics

Data of the total sample demonstrated a sufficient normal distribution of the scores with skewness and kurtosis displayed values around |1|. Table 1 shows the means and standard deviations of the AIDA scores in the subsamples to test for systematic gender and age effects using t-test, calculation of significance *p* and effect size *d*. The score differences between girls and boys were all significant except one (subscale 1.1 with p = .02) but no effect size exceeds the criteria of d > 0.80 to denote a meaningful difference. In contrast, there had been no significant score differences between the younger and the older adolescents, leading to effect sizes about zero. Thus, against our assumptions, data did not support specific group-related developmental stages of identity development.

Analyzing the frequency of T-scores below average (< 40) for the two central *JTCI 12–18 R* character scales, speaking for a high risk of current psychiatric problems, we found 18,1% for Self Directedness and 19,5% for Cooperativeness in this category in the school sample, matching the expected 15–20% of persons showing problems with self-related functionality and social-related adaptability in a typical population sample.

### Criterion validity

We compared the *AIDA* scale and subscale scores between the school sample and the clinical subsample of 20 adolescents with the diagnosis of a personality disorder (18 of them Borderline Personality Disorder) and expected meaningful differences. All scales and subscales differed remarkably between the two groups with effect sizes *d* ranging from 1.04 to 2.56 standard deviations, displaying an excellent discrimination between the patients and the students (see Table 4). The subscales “1.2 Discontinuity-relationships” (d = 2.27) and “2.1 Incoherence-consistent self image” (d = 2.56) showed the strongest discrimination, while “2.2 Incoherence-autonomy” showed the lowest discrimination between the adolescents from school and from the clinical group wit PD.

**Table 4:** **Different mean scores (M) and standard deviations (SD) between the school sample and the clinical subsample with personality disorders (PD) and associated effect size  *d* **

	**M (SD) N = 305school**	**M (SD) N = 20clin-PD**	Effect size d*
AIDA total score:	**65.87 (26.26)**	**129.75 (32.57)**	d = **2.17**
**Identity Diffusion**			
**1. Discontinuity**	27.72 (11.49)	56.20 (14.74)	d = **2.17**
1.1 attributes	12.95 (5.29)	20.75 (7.16)	d = **1.25**
1.2 relationships	6.48 (4.78)	19.65 (6.82)	d = **2.27**
1.3 emotional self refl.	8.30 (4.57)	15.80 (5.95)	d = **1.43**
**2. Incoherence**	38.15 (16.85)	73.55 (19.65)	d = **1.94**
2.1 consistent self	12.65 (7.09)	30.95 (7.20)	d = **2.56**
2.2 autonomy	15.21 (7.37)	24.30 (10.04)	d = **1.04**
2.3 cognitive self-refl.	10.29 (5.14)	18.30 (6.82)	d = **1.34**

* = Significance of all score differences were p < .001.

In contrast to the scale scores, the scores from the six semi-open questions did not differ directly between patients and controls with sufficient effect sizes, e.g. patients did not state less hobbies or peer group affiliations than the students, but some of the evaluative variables derived from the open answers did. The expert-rated variable “sense / compliance” (d = 1.90) and the frequency of giving negative statements for “self” (d = 1.55) and “friend” (d = 0.99) showed remarkable differences between students and patients. While 97.4% of the students gave answers absolutely appropriate to the questions, displaying a high amount of compliance as well as of coherence between asked question and given answer, only 22.4% of the patients did so. 34.7% of the patients gave answers that made “quite appropriate” sense, 26.5% gave responses that were “middle appropriate”, and 14.3% of the patients gave responses that were “quite freestyle” (e.g. giving nonsense answers or describing attributes and experiences when asked about hobbies). Of particular interest, displaying a high amount of self-stigmatizing attitude, is reporting negative attributes or roles (e.g. revengeful, boring, liar, looser) for self or best friend. This happened rarely by the students, (neither for self description (94.1% no negative statements) nor for the best friend (91.3%)), but often in the clinical group. Only 5.9% of the patients did not mention any negative attributes for the self, only 8.7% for the best friend and, therefore, told only positive things in this questionnaire situation.

## Discussion

In the new revision of *DSM-V*, the two core criteria of personality disorders will be significant impairments in “self” and “interpersonal” functioning that are assumed to be continuously distributed. According to this upcoming conceptualization of personality disorders, self-functioning is defined by the two constructs identity and self-direction. Therefore, the reliable, valid, and age-appropriate assessment of identity will be of high interest. Up to now, there is no elaborated self-rating inventory to assess identity development in healthy and disturbed adolescents, so we developed the questionnaire *AIDA* (Assessment of Identity Development in Adolescence) and examined its psychometric properties in referred and non-referred samples.

As identity is a highly complex psychological construct, it was essential to base the new assessment tool on a broad theoretical background, including psychodynamic and social-cognitive theories as well as concepts about identity development. One of our major aims was a source-oriented conceptualization of the construct regarding psychological, social or functional constituents to overcome shortcomings of previous instruments that are mostly based on a phenotypical structure and limited in their focus either on healthy or on disturbed identity development. However, the theory-based approach makes it more difficult to prove psychometric properties of an assessment tool using the customary statistical techniques based on homogeneity and on phenotypical covariations. With “genotypical” models like that, validation with external variables is a key issue, i.e. discrimination between psychiatric categories or between other functional-based or biology-based genotypes.

Taking these challenges into account, the results of our study concerning the psychometric properties of *AIDA* are very promising, for both the adequacy of the derived model of identity as well as for the test construction on item level. Statistical item analysis and selection, based on an initial item pool established deductively by expert consensus and tested in a mixed school and clinical sample to gain optimal data variance, led to a psychometrically sound final version of *AIDA,* with very good scale reliabilities, balanced content consistent with the hypothesized model, and a minimum number of items.

Based on theory, we distinguished the two domains “Continuity” (subjective emotional self-sameness and stability over time) and “Coherence” (cognitive clarity of self-definition and consistency over situations), in line with the constructs´ dichotomy in social-cognitive psychology as well as in the psychopathology-oriented psychodynamic descriptions, to reflect the assumed basic constituents of “Identity Integration vs. Identity Diffusion”. The scales are coded towards psychopathology, thus named Discontinuity and Incoherence, and composed of each three distinct theory-based subscales reflecting basic qualities of psychosocial functioning, covering and reassembling the known subconstructs of identity used in established models, especially Kernberg [21], Westen [22], Fonagy [15], and Akhtar & Samuel [50]. Despite this heterogeneity in content, the good scale reliabilities (i.e. Cronbach’s Alpha which is a measure of internal consistency) argue for a high reliability and, therefore, internal construct validity in terms of statistical homogeneity. With internal consistencies of α = .94 for the total score Diffusion, α = .86 for Discontinuity, α = .92 for Incoherence, and a range of α = .73 – .86 for the subscales, *AIDA* meets the criteria for very good to excellent reliability psychometrically. These results maintained stability in subsamples concerning gender and age, implying a successful item construction that avoids systematic item bias. Adapting to the standard, we analyzed the statistical dimensionality of *AIDA* by using explorative factor analysis (EFA) modeling phenotypical covariations (see above). As expected, the correlational pattern between the *AIDA* items reflected an unspecific phenotype of 15 components with one joint factor combining the most explanation of variance, speaking for the adequateness of using a total score.

The correlational pattern between the *AIDA* scales and subscales, sharing the same higher order construct, reflected a valid internal structure in terms of construct validity. It highlighted both the appropriateness of subdividing the components of identity into subscales as well as using the total summarized scores as a measure of the global construct of identity because the subscales correlated high with their assigned primary scale and lower and with varying amounts with the other primary scale and the subscales. The often mixed phenotypically similar but clinically distinct constructs “stable attributes and goals” (1.1) and “not acting contrary / consistent self” (2.1) only correlated to r = .49. Similarly “stabilizing relationships and roles” (1.2) and “no over-identification / autonomy” (2.2) only correlated to r = .46. This indicates a successful scale construction that avoids trivial conceptual overlap and successfully captures the “psychological genotype” by further subdividing “identity related to self” and “identity related to the social world” along the two areas of identity Continuity and Coherence. Nevertheless, the high correlations (greater than .70) between six of the subscales and especially between the primary scales (.76) are speaking for the adequateness to calculate a meaningful total score for “Identity Integration vs. Diffusion” as well.

The *AIDA* scales showed promising discriminant and convergent validity by meaningful covariations with the *JTCI 12–18 R* personality factors in line with the predictions. We expected the pathology-related personality factors Self Directedness / self-related functionality, Cooperativeness / social-related adaptivity (both character factors) and Harm Avoidance / behavioral inhibition (temperament factor) [56,64] to correlate substantially (>.30) with both *AIDA* scales Discontinuity and Incoherence, both constructed as an indicator for pathology-related identity diffusion. In contrast, we expected only minor correlations with the other temperament factors, seen as closer related to style of behavior than to an impaired personality functioning. As expected, all identity -scales and subscales showed high negative correlations with the *JTCI 12–18 R* character scale Self Directedness (−.59 – -.76), substantial positive correlations with the temperament scale Harm Avoidance (.33 – .60), and only low to moderate correlations (less than .30) with the other scales. But, contrary to our assumptions, only very low correlations with the character scale Cooperativeness (.03 – -.27) occurred. Thus, identity integration seems to be much closer to self-related personality functioning (scale Self Directedness) than to social adaptability (scale Cooperativeness). A remarkable result are the low correlations (.03 -.12) of all identity scales with the temperament factor Novelty Seeking that (in part) represents impulsive behaviour. This is a clear indication that in contrast to other identity questionnaires our operationalization of identity successfully kept out trait impulsivity. Similarly, the low correlations between the *AIDA* subscale “Incoherence-autonomy vs. over-identification” and the temperament factor Reward Dependence / social responsiveness (−.01) and the character factor Cooperativeness (.00) may speak for the successful attempt to avoid trivial item overlap between alternative constructs in general and an overlap with sociability in particular.

Little is known about the development of identity over time and if the process of identity formation is different for girls and boys. According to our data, we can assume that the way in which younger adolescents describe their identity using the *AIDA* items is not much different from that of older adolescents (no significant differences, effects sizes around zero). Although, of course, adolescents do differ and develop in terms of identity integration with age, there seemed to be no systematic “normative” age levels and no typical developmental stages per age could be found. Therefore we can assume that identity development as it is measured by *AIDA* reflects age neutral Identity Integration vs. Identity Diffusion. Thus, separate population norms would be redundant for age groups. In contrast, the differences between girls and boys were significant in all scales and subscales on the 1% level, except subscale 1.1 (Discontinuity concerning attributes and goals) with medium effect sizes for the primary scales (.45 and .56). The medium effect size is large enough to warrant continued separation of gender at this stage of instrument development, until further data from the studies currently underway is analyzed.

Our approach to integrate some semi-open questions to simulate an interview-like situation and to catch some additional facets of identity diffusion did succeed partly and is not fully explored in its potential yet. It delivered at least one valid additional content that is integrated in Westen’s concept [22] but missing on the traditional item level in *AIDA*, the concept of “self-stigmatizing” which is described as a sign of disturbed identity development. The frequency of “giving negative statements” for self and best friend seems to differentiate remarkably between students and patients in general, as the students rarely (under 10%) labeled themselves or their best friend in negative terms (e.g. “a looser”), while the patients did so frequently (over 90%). But the significance of these results concerning the evaluation of adolescent identity integration needs further investigation.

As we have outlined above, disturbance of identity is seen as one of the core components of of personality disorders. Therefore, an instrument that is designed to capture disorders of identity in adolescents should have the ability to distinguish between normal adolescents and those who suffer from a personality disorder. Criterion validity was achieved, as the two *AIDA* primary scales, as well as all subscales, revealed an excellent discrimination between patients with personality disorders and normal controls with effect sizes (d) between 1.04 and 2.56 standard deviations. The subscales “stable relationships and roles” (d = 2.27) and “consistent self concepts” (d = 2.56) differed the most (comparable to an IQ difference of 85 to 122.5), while the subscale “autonomy vs. over-identification” differed (though above criteria) the least (d = 1.04) between adolescents with and without PD. Distinct relationships between subconstructs of identity development and different diagnoses will be of continued interest. Future studies will have to explore the effectiveness of *AIDA* to detect changes in identity integration as identity consolidation is one of the major aims of psychotherapy with personality disordered adolescents.

### Limitations

We did not assess psychiatric disorders in the school sample. But, with respect to the results of epidemiological studies, we can assume that up to 15–20% of this adolescent sample of the general population show minor to major signs of mental problems. The frequency of T-scores below average (< 40) for the *JTCI 12–18 R* character scales Self Directedness (18,1%) and Cooperativeness (19,5%) in the school sample gives support for this assumption and, thus, a successful study design with a representative population sample, though a personality inventory can never be the sole basis for a psychiatric diagnosis. Moreover, this gives rise to the expectation that the differences in the *AIDA* scores between our clinical group and a completely healthy control group would be even higher.

Further research and the comparison of developmental stages and pathways into adulthood between school samples from different countries and clinical samples with different diagnoses or special developmental problems will be of high interest concerning not only the criterion validity but also the construct validity of *AIDA*. With 52 adolescents, the clinical sample was too small and heterogeneous to build more sufficiently large diagnose-related groups for e.g. Eating Disorders or Conduct Disorders and to analyze systematic differences in *AIDA* results between them. Additionally, test-retest reliability was not measured and should be examined in further studies.

The scale structure and its subdivision reflects the theory-based “genotype” of the complex construct “Identity Integration vs. Identity Diffusion” in terms of the assumed psychological, social and functional constituents and should be seen as a summary of all relevant subconstructs. However, additional studies are needed to address the genotypical approach not only concerning psychosocial but also possible biological constituents (i.e. biological markers of personality disorders).

Nearly all theoretically described contents could be kept in the scales in a balanced way. However, some contents, especially all items reflecting sexuality and other potentially embarrassing issues, could not be kept because of dramatically weak psychometric properties. This is a sign of non-adequacy and non-applicability of these contents in the form of a self-report item and should not be taken as a sign of unimportance of these facets concerning identity development. To the contrary, for clinical evaluation these contents should be assessed, but within a personal therapeutic relationship. Similarly, the breadth of the integrated contents, especially on subscale and facet level, should not be overinterpreted. For example the facets “autonomous affect-regulation” and “subjective self-sameness” are each represented by only two power-items, and thus adequately representative in our very condensed model of identity but, of course, are not described in an exhaustive way.

## Conclusion

The present data suggest that *AIDA* is a reliable and valid instrument to assess normal and disturbed identity in adolescents and discriminates well between patients with PD and healthy controls. It was designed based on a broad range of theoretical approaches from the field and in a joint international project with expert consensus, focussing on a deductive scale construction, on clarity, culture, and -age, and -gender fairness of the items. Thus, studies addressing the sources of behavior or personality disorders as well as studies comparing identity development in different countries or adolescent subsamples remains of high interest. Moreover, development of identity over time should be analyzed with longitudinal approaches, as it does not seem to be simply related to age. Several translation and validation studies for *AIDA* (Chile, Brazil, Mexico, Spain, Canada, Kosovo, Croatia, Bulgaria, Serbia) as well as studies for further validation with detailed analyses of covariation with personality development on the subscale level, of discrimination between distinct psychiatric disorders like anxiety, attention, and eating disorders in contrast to PD and for providing population norms, are already in progress in cooperation with the authors.

## Competing interests

The authors declare that they have no competing interests.

## Authors’ contributions

KG, PF, SS and KS developed AIDA. KG designed the study, performed the statistical analysis and was the main writer of the manuscript. KS and PF wrote parts of the manuscript. EJ, OP, MB and SS collected the data. All authors read and approved the final manuscript.
